# Neural Correlates of Contrast and Humor: Processing Common Features of Verbal Irony

**DOI:** 10.1371/journal.pone.0166704

**Published:** 2016-11-16

**Authors:** Alexandre Obert, Fabien Gierski, Arnaud Calmus, Aurélie Flucher, Christophe Portefaix, Laurent Pierot, Arthur Kaladjian, Stéphanie Caillies

**Affiliations:** 1 C2S Laboratory (EA 6291), University of Reims Champagne-Ardenne, Reims, France; 2 Department of Psychiatry, Robert Debré Hospital, Reims University Hospital, Reims, France; 3 Department of Physical Medicine and Rehabilitation, Sébastopol Hospital, Reims University Hospital, Reims, France; 4 Department of Medical Imaging, Maison Blanche Hospital, Reims University Hospital, Reims, France; 5 CReSTIC Laboratory (EA 3804), University of Reims Champagne-Ardenne, Reims, France; University of Nottingham, UNITED KINGDOM

## Abstract

Irony is a kind of figurative language used by a speaker to say something that contrasts with the context and, to some extent, lends humor to a situation. However, little is known about the brain regions that specifically support the processing of these two common features of irony. The present study had two main aims: (i) investigate the neural basis of irony processing, by delivering short ironic spoken sentences (and their literal counterparts) to participants undergoing fMRI; and (ii) assess the neural effect of two irony parameters, obtained from normative studies: degree of contrast and humor appreciation. Results revealed activation of the bilateral inferior frontal gyrus (IFG), posterior part of the left superior temporal gyrus, medial frontal cortex, and left caudate during irony processing, suggesting the involvement of both semantic and theory-of-mind networks. Parametric models showed that *contrast* was specifically associated with the activation of bilateral frontal and subcortical areas, and that these regions were also sensitive to *humor*, as shown by a conjunction analysis. Activation of the bilateral IFG is consistent with the literature on humor processing, and reflects incongruity detection/resolution processes. Moreover, the activation of subcortical structures can be related to the reward processing of social events.

## Introduction

Verbal irony is a kind of nonliteral language that is traditionally viewed as a figure of thought, in the sense that the semantics of an ironic sentence and a nonironic one are indistinguishable [1]. This feature means that context plays a particularly important role in narrative comprehension. For example, in order to understand the comments “He is bright” about an idiot or “He is stupid” about somebody intelligent, it is necessary to take into account the reality of the situation, generally named the *context*. In most theories, verbal irony comprehension relies on an incongruity resolution process, following the detection of an incongruity (termed *contrast* in the irony literature) between the assertion (i.e., ironic sentence) and the reality (i.e., context) [2]. According to prevailing theory, humor relies precisely on the detection of this contrast, which produces a violation of expectations and/or facts that needs to be resolved[3]. It is further generally acknowledged that irony is used because of its humorous effect[4]. There have even been suggestions that irony serves a social function, enabling us to say something negative with humor, and thus attenuate the negative impact on the speaker-listener relationship [5]. Despite this, very few studies have explicitly explored the neural bases of both the contrast effect and humor appreciation in irony comprehension.

Most findings regarding contrast and humor come from rating studies. The groundbreaking research by Colston and O’Brien [6] highlighted the importance of the contrast effect on pragmatic goals of verbal irony such as humorousness. These authors found that ironic sentences were rated as more contrasted and more humorous than their literal counterparts, and that the pragmatic goal of humor was influenced by the perceived contrast. Moreover, in a second study[7], they found that making positive comments about negative events (e.g., verbal irony) was more likely to involve humor than exaggerating these events (e.g., hyperbole), and strongly negative comments (strong irony) had greater pragmatic effects than weaker negative comments (weak irony). Similarly, when Ivanko and Pexman [8] modulated the contrast between context and critical assertion by accentuating the negativity of the context, they found that a greater contrast appeared to heighten the perception of sarcasm (see also [9]), which is traditionally viewed as a kind of ironic statement where ridicule is an important component [10]. In a recent study, however, Calmus and Caillies [2] showed that contrast (between the context and what is said) and humorousness are linked in a nonlinear manner, in an inverted U-shape, such that the funniest sentences are those with moderate contrasts between the context and the ironic assertion. All these studies therefore emphasize the role of the contrast effect in ironic humor.

Even today, little is known about the brain network that subtends the processing of these two interconnected features when dealing with irony. Relatively few studies have examined the cerebral bases of irony processing in healthy participants using functional magnetic resonance imaging (fMRI). Some of these have reported areas of activation, which are discussed in the light of theory of mind (ToM; drawing of inferences about others’ mental states and intentions) and semantic processes. Studies have shown activation of the medial prefrontal cortex [11–13], left and right temporoparietal junction (TPJ; [13,14]), and superior temporal sulcus (STS; [12]) (STS; 12), which are recognized as forming part of the ToM network. As well as this ToM network, some authors have identified a broad language network dedicated to semantic and contextual processing. Eviatar and Just [15] showed that the right superior and middle temporal gyri (MTG) are more likely to be activated by ironic sentences than by literal ones. These authors interpreted this as the neural expression of two kinds of (nonexclusive) processes: the ability to grasp the speaker’s communicative intent; and the integration of different items of information into a single, coherent story. Similarly, using sarcastic sentences, Uchiyama et al. [16] observed left temporal activation for processing both the context and the critical ironic sentence, supporting the idea of a general involvement of left temporal areas in contextual and semantic processing. This is congruent with some other studies providing evidence that the temporal gyri are involved in inference processing [17] and semantic incongruity [18]. Additionally, Uchiyama et al. [16] reported activation of the left inferior frontal gyrus (IFG) during the processing of sarcasm, and argued that it must be a key region for integrating ToM and semantic processes. Furthermore, a recent meta-analysis by Rapp et al. [19] demonstrated that the lIFG is a core region for several types of nonliteral language, including metaphor, idiom and irony. This can be explained by the higher cognitive demands (in terms of semantic processing) made by figurative expressions, and by the fact that the lIFG has been shown to be involved in several language processes, including semantic integration [20,21]. Finally, similar areas of activation have been reported in studies of incongruity based on the detection-resolution model of humorous stimuli. Samson, Zysset, and Huber [22] showed that the temporoparietal junction is activated during the processing of cartoons requiring ToM processes, and suggested that this reflects successful incongruity resolution processing, whereby complex information is integrated into a coherent meaning [3,23,24].

Taken together, these results shed some interesting light on the semantic processes underlying irony comprehension, and thus on the semantic resolution of the incongruity resulting from the contrast between remark and context. However, very few of them concern the neural bases recruited by the humorous aspect of ironic sentences. To our knowledge, the only fMRI study to have included an analysis of humor appreciation is Akimoto et al. [25]’s study. After the scanning session, participants were asked to rate the stimuli on perceived humor, in addition to the degree of irony. Analyses showed that the right amygdala and hippocampal structures were more likely to respond positively to the degree of irony, and the right dorsolateral prefrontal cortex to the degree of perceived humor. The former were assumed to be sensitive to the emotional states of ironic communication, and the authors postulated that the latter subtends the executive functions involved in the resolution of incongruity, as well as in the social function of irony. These results have been echoed in fMRI studies of humor featuring materials other than verbal irony, namely humorous cartoons or stories. For example, Bartolo et al. [26] observed that the left amygdala was positively linked to the degree of amusement derived from humorous cartoons, supporting the idea that this is a key area for emotional responses to humorous events. For their part, Chan et al. [27] used verbal funny stories containing a situational context (e.g., a child has to share some donuts with his brother) and ending with an unexpected punchline (e.g., “Hey, we have a donut to share! I'll take the circle, and you can have the hole!”). When they compared highly funny stories with less funny ones, the authors observed activation of the left ventromedial prefrontal cortex, along with subcortical activation in the bilateral parahippocampal and amygdala structures. These structures are known to form part of the mesolimbic reward system, which has been shown to be activated by subjective appreciation of humor [28]. Moreover, incongruity resolution and humor processing appear to share common cortical areas. Bekinschtein, Davis, Rodd, and Owen [29] conducted an fMRI study in which they asked participants to rate the funniness of sentences that varied on humorous and contrast features. Their results suggested that resolving the incongruity of these sentences engaged the frontotemporal network, while perceiving their humor involved the subcortical reward network. More interestingly, they observed that the left IFG was recruited by both incongruity resolution and humor processing, confirming that incongruity and humor are strongly linked.

The aim of the present study was to investigate the neural bases of auditory verbal irony comprehension using fMRI, and to specifically assess the responsiveness of different brain areas to contrast and humor features, indirectly taking into account the nonlinear relation between the two that had previously been demonstrated. We reasoned that if contrast affects humor appreciation in a nonlinear manner, the brains regions recruited by contrast would respond quadratically to humor. In our design, the stimuli were presented in the auditory modality, as intonation is an important cue for detecting the contrast between context and critical remark, and thus for understanding the irony [30,31].

## Material and Methods

### Participants

Initially, 23 healthy participants were included in the study. Two of them, however, were excluded owing to poor-quality behavioral data (see below). All the remaining participants (10 women and 11 men; mean age = 22.10 years, *SD* = 1.92, range = 20–27) were native French speakers and right-handed, according to the Edinburgh Handedness Inventory [32]. They had normal or corrected-to-normal vision and normal hearing, and no past or present medical, psychiatric or neurological illnesses. They also met health and safety regulations regarding the use of MRI. The study was designed in accordance with the Declaration of Helsinki and approved by the East III institutional review board of Nancy University Hospital. All participants gave their written informed consent after receiving a full description of the study.

### Stimuli

**Fig 1:**
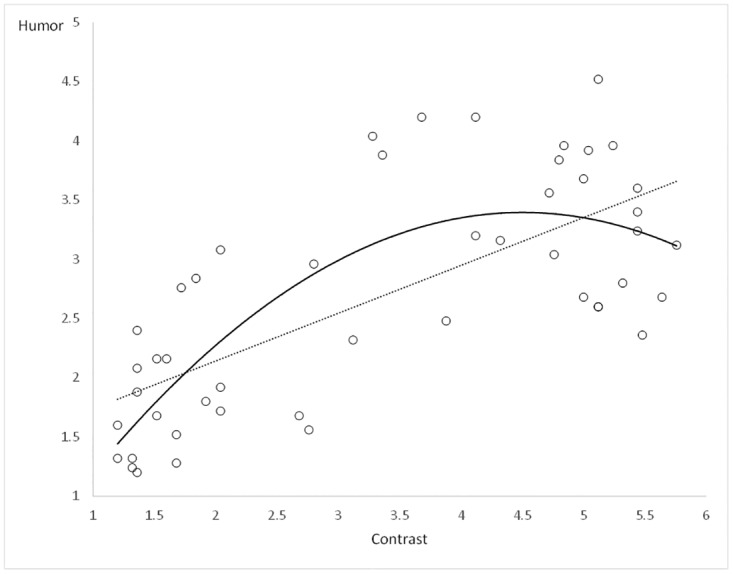
Scatterplot of contrast and humor degrees showing the linear (dash line) and quadratic (solid line) relationships.

Forty-eight two-sentence ironic or literal stimuli were taken from a pool of 74 sentences used in a previous published study [2]. Each ironic story was matched with a literal control story in which the context sentence had been modified in such a way that the second sentence of the pair was no longer ironic (Table 1). It should be noted that the ironic comments were invented, and as a consequence, were neither familiar nor conventional, although they were selected as being easily understandable. Contrast detection and humor appreciation were operationalized with contrast and funniness rating scales. For the contrast judgments, 153 participants were asked to rate the extent to which the meaning of the first sentence contrasted with that of the second sentence on a 6-point Likert-type scale ranging from 1 (*Not at all contrasting*) to 6 (*Highly contrasting*). For the humor judgments, 99 participants (none of whom participated in the contrast judgments) were asked to rate the funniness of the stories on a similar 6-point scale ranging from 1 (*Not at all funny*) to 6 (*Very funny*). For the purpose of the present study, the 24 ironic sentences were all moderately contrasted (*M* = 4.84, *SD* = 0.69) and moderately humorous (*M* = 3.43, *SD* = 0.61), and their literal controls were those with the lowest contrast (*M* = 1.89, *SD* = 0.69) and humor (*M* = 1.96, *SD* = 0.57) ratings. A statistical *t* test for independent samples revealed significant differences between the ironic and literal sentences on both contrast, *t*(46) = 14.80, *p* < 0.001, and humor, *t*(46) = 8.65, *p* < 0.001, with the ironic sentences being judged as more contrasted and more humorous than the literal ones. In order to test the relationship between the degrees of contrast and humor of the selected sentences, linear and nonlinear relations were modeled. At a descriptive level (see Fig 1), the degrees of contrast and humor of the selected sentences were more likely to follow a quadratic distribution than a linear one. Computation of *R*-squared confirmed this view with the quadratic model explaining more variance than the linear one (*R*^2^_quadratic_ = 0.581 > *R*^2^_linear_ = 0.492).

**Table 1:** Examples of the Two-Sentence Stories.

	Example 1	Example 2
Ironic	*C’est la meilleure des promotions que j’ai eues dans cette entreprise*. *Demain*, *je la quitte définitivement*.	*Ce nouveau spectacle n’est pas mauvais*. *C’est tout simplement un navet*.
	(This is the best promotion I’ve ever had in this company. Tomorrow, I’m leaving for good.)	(This new show isn’t bad. It’s complete rubbish.)
Control	*C’est la pire des situations que j’ai eues dans cette entreprise*. *Demain*, *je la quitte définitivement*.	*Ce nouveau spectacle est de mauvais goût*. *C’est tout simplement un navet*.
	(It’s the worst post I’ve ever held in this company. Tomorrow, I’m leaving for good.)	(This new show’s in bad taste. It’s complete rubbish.)

The two-sentence stories were recorded in audio files. Each story was accompanied by a *yes/no* visually presented question concerning its ironic or literal nature. A training session featured two other ironic stories and their literal counterparts.

### Procedure

Participants were invited to listen to the 48 two-sentence stories, which were presented in a semirandom order (fixed order for each participant). A black screen with a fixation point was presented first, via a mirror. It was displayed for between 0 and 3000 ms to introduce jitter. The presentation of this black screen was used to establish a baseline activation level for the first-level functional computations (see below). While participants listened to each sentence pair, a similar and indistinguishable black screen with a fixation point remained in full view. After they had heard the item, another black screen appeared, this time featuring a question mark and the words *ironic* (bottom lefthand corner) and *literal* (bottom righthand corner). Participants were invited to judge the nature of the item and indicate their choice by pressing the corresponding button.

### Imaging procedures

The fMRI data were collected using a Philips Achieva 3.0T whole-body MRI scanner (Philips Medical Systems, Best, Netherlands). The study was performed with a 2D T2*-weighted EPI sequence, in ascending slice order (TR = 2000 ms, TE = 33 ms, 90° flip angle, FOV = 240 mm). Thirty-two slices were acquired, and each slice was 4.5 mm thick, with a gap of 0 mm. The acquisition matrix was 64 x 64 voxels. The functional acquisition sequence was composed of 245 volumes, corresponding to the continuous acquisition of the 32 axial slices, and lasted 8 minutes and 10 seconds. For each participant, we also acquired a high-resolution 3D T1-weighted anatomical image (TR = 8.2 ms, TE = 3.7 ms, 8° flip angle, FOV = 240 mm), allowing us to register the functional data. One hundred and sixty slices were acquired, and each slice was 1 mm thick, with no gap. Structural and functional images were taken in the axial plane parallel to the anterior-posterior commissure (AC-PC) line.

Functional data were preprocessed with Statistical Parametric Mapping (SPM) software Version 8 (Wellcome Department of Cognitive Neurology, Institute of Neurology, London, UK), implemented in MATLAB^®^. Functional images were realigned to the mean functional image of the series, then normalized with reference to a standard brain template (EPI template of the Montreal Neurological Institute, MNI). Data were spatially smoothed using an 8-mm full width at half maximum (FWHM) Gaussian filter. High-pass and low-pass filters were also used, in order to filter the data for artifacts of a physiological origin (breathing, heartbeat).

### Whole-brain analysis

We began by modeling the onset of the correctly classified ironic and literal target sentences as two regressors. The context sentences, fixation crosses and participants’ response events were also modeled across these two conditions, leading to three additional regressors. In the first analysis, we compared the ironic condition with the literal one.

In order to test the effects of the contrast and humor features of irony on brain activity, taking account of the nonlinear effect of contrast on humor appreciation [2], we calculated two parametric models. As indicated earlier, our reasoning was that if contrast affects humor appreciation in a nonlinear manner, then brain regions affected by contrast should respond quadratically to the humor. A first parametric model was calculated using all the correctly classified sentences as a single regressor and contrast values as parametric modulator with a negative polynomial quadratic function [33]. Context sentences, fixation crosses and participants’ response events were entered as covariates of no interest. A second design contained the same regressors, but with humor as a parametric modulator with a linear polynomial function. For both designs, contrast images representing the effects of contrast and humor were computed for each participant. In order to identify the brain regions that responded to both contrast and humor features, we performed a conjunction analysis with a conjunction null hypothesis, using these individual contrast images [34]. For the ironic versus literal comparison, parametric models and conjunction analysis, activation maps were thresholded at *p* = 0.001 uncorrected, with a cluster extent of 20 voxels, and labelled using the AAL toolbox for SPM8 [35].

## Results

### Behavioral results

We used Grubbs’ method to assess outliers for both accuracy and response times. Two participants were excluded because they provided fewer correct responses than the group as a whole. We ran two one-way repeated-measures analyses of variance (ANOVAs) on the mean number of correct responses as a function of condition (ironic vs. literal): one per participant (*F*_1_), the other per item (*F*_2_). The mean number of correct responses in the ironic condition did not differ significantly from that in the literal condition, *F*_1_(1, 20) = 1.74, *p* = 0.20, and *F*_2_(1, 23) = 0.40, *p* = 0.53. Similar results were observed for mean correct response times, *F*_1_(1, 20) = 1.25, *p* = 0.28, and *F*_2_(1, 23) = 0.25, *p* = 0.53 (Table 2).

**Table 2:** Behavioral data per participants and per items.

Conditions	Reaction times (ms)		Correct responses	
	(Mean ± SD)		(Mean ± SD)	
	Participants	Items	Participants	Items
**Irony**	1008.75 ± 234.79	1014.46 ± 200.21	20.99 ± 1.78	20.99 ± 1.78
**Literal**	953.23 ± 262.23	989.69 ± 257.76	20.18 ± 2.38	20.18 ± 2.38

### Brain activation results

#### Ironic versus literal conditions

**Fig 2:**
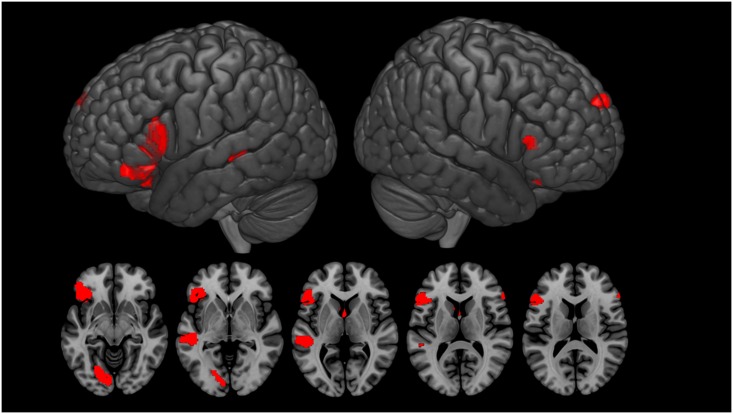
Activation for the Irony > Literal comparison. *p* < 0.001 uncorrected, k = 20 voxels. Differences were observed in the bilateral inferior frontal gyrus, left superior temporal gyrus, lingual gyrus caudate, and bilateral medial superior frontal gyrus.

The ironic minus literal comparison revealed a frontotemporal pattern of activations: in the posterior part of the left superior temporal gyrus (STG), encompassing the superior temporal sulcus (STS), as well as the bilateral inferior frontal gyrus (IFG), left caudate, and left lingual gyrus extending to the fusiform area (Table 3 and Fig 2).

**Table 3:** Whole-Brain Activation for the Irony > Literal Comparison.

Location				Significance		MNI coordinates		
Area	Hemisphere	BA	No. voxels	*p*_FWE-corr_	*t* values	x	y	z
Superior temporal gyrus	L	21	325	0.016	6.73	-49	-29	1
Inferior frontal gyrus	L	45	1079	0.001	6.47	-51	23	11
Lingual gyrus	L	18	565	0.001	6.05	-11	-83	-9
Medial superior frontal gyrus	R	10	31	0.875	4.58	9	59	29
Inferior frontal gyrus	R	45	35	0.845	4.58	59	29	11
Caudate	L	-	61	0.632	4.50	-1	7	7
Medial superior frontal gyrus	L	10	49	0.731	4.31	-5	61	31
Inferior frontal gyrus	R	38	20	0.944	4.17	45	29	-21

*Note*. L = left; MNI = Montreal Neurological Institute; *p*_FWE-corr_: *p* = 0.05 cluster threshold corrected for familywise error. *p* < 0.001 uncorrected; k = 20.

#### Effects of contrast and humor

**Fig 3:**
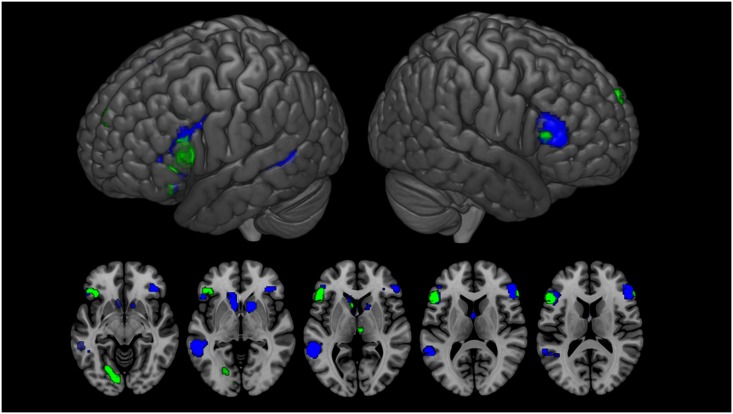
Activation for the Contrast (blue) and Humor (green) effects. *p* < 0.001 uncorrected, k = 20 voxels. Common activations were localized in the bilateral inferior frontal gyrus and left caudate.

A negative quadratic effect of contrast was observed in the bilateral IFG, extending to the middle frontal gyrus (MFG) in the left hemisphere. Left activations were also observed in the superior parietal gyrus and posterior part of the MTG encompassing the STG and STS. Activations in the right medial frontal gyrus (MedSFG), extending to the supplementary motor area and bilateral subcortical regions, specifically the caudate and putamen, were also significant. A linear effect of humor was observed in the bilateral IFG, anterior part of the MedSFG, right thalamus and left caudate. Additionally, activations in the left lingual gyrus were recorded (Table 4 and Fig 3). The conjunction analysis revealed that the bilateral IFG and left caudate were both significantly activated by contrast and humor (Table 4).

**Table 4:** Whole-Brain Activation for Contrast and Humor.

Location				Significance		MNI coordinates		
Area	Hemisphere	BA	No. voxels	*p*_FWE-corr_	*t* value	x	y	z
*Contrast*								
Inferior frontal gyrus	R	45	525	0.001	7.58	57	37	15
Middle temporal gyrus	L	21	563	0.001	7.46	-59	-45	1
Medial superior frontal gyrus	R	8	720	0.001	7.27	3	25	49
Inferior frontal gyrus	L	48	844	0.001	6.23	-47	17	25
Caudate	L	25	232	0.020	5.92	-9	23	-1
Putamen	R	-	124	0.157	5.79	15	9	-3
Middle temporal gyrus	L	39	36	0.822	4.85	-41	-53	19
Inferior frontal gyrus	R	47	106	0.226	4.72	41	35	-7
Middle frontal gyrus	L	6	24	0.929	4.24	-17	5	53
Middle temporal gyrus	L	20	23	0.936	4.23	-47	-43	-11
Superior Parietal Gyrus	L	7	26	0.914	4.04	-25	-75	41
Inferior frontal gyrus	L	45	21	0.949	4.01	-45	39	7
*Humor*								
Inferior frontal gyrus	L	48	587	0.014	7.31	-49	21	17
Inferior frontal gyrus	R	45	31	0.875	5.46	61	31	11
Lingual Gyrus	L	17	238	0.047	5.16	-11	-83	-9
Medial superior frontal gyrus	L	10	60	0.636	4.52	-5	61	31
Thalamus	R	-	21	0.939	4.17	5	-19	5
Caudate	L	25	22	0.934	3.91	-7	15	5
*Conjunction between Contrast and Humor*								
Inferior frontal gyrus	L	48	346	0.012	4.90	-49	21	19
Inferior frontal gyrus	R	45	28	0.456	4.43	61	31	11
Caudate	L	25	34	0.971	3.67	-9	17	-1

*Note*. L = left; MNI = Montreal Neurological Institute; *p*_FWE-corr_: *p* = 0.05 cluster threshold corrected for familywise error. *p* < 0.001 uncorrected; k = 20.

## Discussion

The present study used fMRI to assess the neural bases of the comprehension of auditory verbal irony, more specifically the brain areas involved in irony processing as a function of contrast and humor features. Results showed that, compared with the processing of literal stimuli, irony processing was associated with specific patterns of activation, the most robust activations being observed in the posterior part of the left STG and left STS, as well as in the left IFG. Additional activations were observed in the right IFG and caudate structure. More interestingly, we found that contrast, acting quadratically, also triggered activations in these regions, as well as in the left MFG and putamen. A linear effect of humor was observed in the bilateral IFG, medial SFG, right thalamus, left caudate and left lingual gyrus, with the strongest activation in the IFG. Lastly, analysis of the conjunction between contrast and humor showed that the greatest significant common activation was located in the left IFG, with additional common activation in its right homologue, as well as in the left caudate.

### Activations during irony processing

As previously mentioned, our irony versus literal comparison highlighted activations in the posterior part of the left STG and left STS, as well as the caudate, bilateral IFG and medial SFG that were specific to the processing of ironic sentences. This pattern of activations can be attributed to both theory of mind (ToM) and semantic processes.

Irony is often regarded as a figure of thought that requires mentalizing processes. Many studies have been designed to investigate this specific question, and these have consistently found activation of the brain areas recruited by ToM, such as the superior temporal sulcus and medial SFG [11,12,16]. These areas are part of a well-known ToM network [36,37] that is strongly elicited during irony processing, as the latter requires higher-order inferences about others’ beliefs and intentions [38]. The activation of the IFG, and more particularly the left IFG, which appears more robust, is consistent with studies revealing that this region plays a semantic role in figurative language processing, particularly the left IFG. For instance, Rapp et al. [19] observed left IFG activation for all the figurative expressions they studied (metaphors, idioms, metonymy and irony). According to these authors, this region is involved in contextual integration, meaning selection and evaluation, and integration of world knowledge into sentences. In their meta-analysis, Vigneau et al. [39] also reported robust activation of the left IFG for both semantic and sentence processing, as well as the left STG for sentence processing. Taken together, the left STG and left IFG activations appear to confirm the hypothesis that semantic integration processes are engaged in irony comprehension. One potential issue regarding this interpretation concerns the modality of stimulus presentation in our study, as we cannot exclude the possibility of an overlap in the areas associated with auditory or semantic processing. Indeed, Frühholz and Grandjean [40] suggested that the STG, along with the bilateral IFG, is involved in affective prosody processing. Consistent with this, Matsui et al. [41] showed bilateral IFG activation when processing ironic prosody. Following these authors, the right IFG would be sensitive to the detection of statement-prosodic incongruity, while the left IFG is engaged more during information selection and integration, as required in case of disparity between context and sentence meaning.

Furthermore, ToM and semantic language processes may interact during irony processing, instead of being mutually exclusive. When Kuperberg et al. [42] compared sentences containing a pragmatic, semantic or syntactic violation with normal ones, they found that the left STG was activated more for pragmatically violated sentences than for semantically or syntactically violated ones. The authors interpreted this as reflecting reevaluation of the sentence when the pragmatic meaning could not easily be grasped. Moreover, based on evidence of STS activation in both language and mentalizing tasks, Redcay [43] suggested that the superior temporal area endorses the analysis of visual and verbal inputs and the extraction of their social meanings. In the same vein, Spotorno et al. [13] showed that parts of the medial frontal cortex and left IFG strongly interact during irony processing, suggesting information exchanges between ToM and linguistic networks. Taken together, these findings suggest that auditory irony processing engages areas dedicated to the integration of linguistic auditory inputs, which require linguistic and mentalizing processes to grasp their socially consistent meaning.

In studies of language processing, the caudate has been identified as a key region in meaning selection and ambiguity resolution [44,45]. This process is probably needed more to *get the joke* with verbal irony, as this figure of speech can convey the opposite of what is being said. However, very few studies of irony have reported subcortical activation, and this kind of activation has therefore seldom been discussed [14,46,47].

### Neural correlates of contrast and humor

The results modulated by the contrast revealed activation of left areas in the IFG, MFG, MTG extending to the STG, and parietal gyrus. Activations in the right medial SFG and bilateral caudate and putamen were also observed. A linear effect of humor was observed in the bilateral IFG, medial SFG, right thalamus, left caudate, and left lingual gyrus. Finally, the bilateral IFG and left caudate appeared to be sensitive to both the contrast and humor features.

The left IFG had previously been highlighted in studies of humor processing. According to Gardner et al. [48], humorous events are processed in two stages: humor detection and humor appreciation. The purpose of the first stage (cognitive component) is to understand the disparity between the unexpected ending and the situation. The second stage corresponds to the affective component, and is responsible for the emotional enjoyment of the humorous event (see also [49] for a complementary perspective). In line with this theory, Moran et al. [50] found that the left IFG reacted to the first component (i.e., humor detection) when participants were asked to watch episodes of sitcoms. Chan et al. [27], who tried to segregate these two components of humor processing using verbal stories, also observed activation of the bilateral IFG when participants were engaged in a humor comprehension process. Similarly, Bartolo et al. [26] found that the left and right IFG were activated more during the processing of funny versus unfunny cartoons, and the right IFG was also positively correlated with the feeling of amusement. Thus, the authors suggested that while the left IFG is involved in incongruity detection, its right homologue is engaged in the incongruity resolution that is required to achieve a feeling of amusement. Thus, the bilateral IFG could be a key region for incongruity detection and resolution when processing humorous events.

The explanation for the involvement of subcortical structures in irony processing remains unclear. Activation of the caudate nucleus and putamen could be related to the decision-making process inherent to the task, and reflect the activation of the *executive* frontal-subcortical loops [51]. However, when focusing on the potential effect of humor in different kinds of stimuli, humor can be seen to involve the activation not just of cortical areas, but also of subcortical ones that are known to be associated with the reward system [27,50]. This system constitutes a network encompassing both cortical (e.g., medial temporal, prefrontal and orbitofrontal cortices) and subcortical areas, including midbrain structures (ventral tegmental area, substantia nigra) and the ventral striatum (nucleus accumbens, caudate, putamen) [52]. Data from the literature show that this system is activated during the processing of humorous stimuli such as cartoons [26,53], comedy clips [54], and verbal jokes [29], and could be thus extended to ironic sentence listening.

One possible caveat of the present study may lie in the methodology we used, as the humor ratings were not provided by the participants themselves, but were taken from an earlier stimulus validation. Unlike contrast, which more strongly refers to a feature of the stimuli, humor appreciation is a very individual experience. The pattern of activation we observed may not, therefore, directly relate to individual differences in irony. As a consequence, we acknowledge that further studies will be needed to confirm our results.

Furthermore, it may be important to distinguish humor from other ways of triggering emotional states. The ironic material used in studies frequently singles out a specific victim for differing degrees of mockery [14,15,25]. However, irony and sarcasm have been shown to induce different emotional states and different emotional attributions, with sarcasm producing more negative feelings in addressees [55]. Future studies could therefore be conducted to investigate the specific neural correlates associated with these different rhetorical devices.

Taken together, the results of the present study highlight the brain network involved in the processing of auditory ironic sentences. They also reveal that some brain regions that are sensitive to the processing of the disparity between context and critical sentence are also sensitive to the humor intensity of the sentences. Our results echo the literature findings on humor, and underline the importance of both cortical and subcortical structures. Moreover, some components of the reward system have been found to be involved in the processing of humorous events, supporting the notion that both social and emotional processes are engaged. Nevertheless, further studies are needed to confirm the involvement of these structures and investigate whether this system is linked to the successful decoding of irony or to the processing of the stories’ degree of humor. Furthermore, our results suggest that the lIFG is a key region that supports both contrast and humor features through an incongruity detection-resolution process.
